# Marine-Bioinspired Nanoparticles as Potential Drugs for Multiple Biological Roles

**DOI:** 10.3390/md20080527

**Published:** 2022-08-18

**Authors:** Geum-Jae Jeong, Sohail Khan, Nazia Tabassum, Fazlurrahman Khan, Young-Mog Kim

**Affiliations:** 1 Department of Food Science and Technology, Pukyong National University, Busan 48513, Korea; 2 Department of Biotechnology, Jaypee Institute of Information Technology, Noida, A-10, Sector-62, Noida 201309, Uttar Pradesh, India; 3 Industry 4.0 Convergence Bionics Engineering, Pukyong National University, Busan 48513, Korea; 4 Marine Integrated Biomedical Technology Center, The National Key Research Institutes in Universities, Pukyong National University, Busan 48513, Korea; 5 Research Center for Marine Integrated Bionics Technology, Pukyong National University, Busan 48513, Korea

**Keywords:** marine, nanoparticles, infectious disease, antimicrobial, anticancer, antioxidant, antiinflammatory, antidiabetic

## Abstract

The increased interest in nanomedicine and its applicability for a wide range of biological functions demands the search for raw materials to create nanomaterials. Recent trends have focused on the use of green chemistry to synthesize metal and metal-oxide nanoparticles. Bioactive chemicals have been found in a variety of marine organisms, including invertebrates, marine mammals, fish, algae, plankton, fungi, and bacteria. These marine-derived active chemicals have been widely used for various biological properties. Marine-derived materials, either whole extracts or pure components, are employed in the synthesis of nanoparticles due to their ease of availability, low cost of production, biocompatibility, and low cytotoxicity toward eukaryotic cells. These marine-derived nanomaterials have been employed to treat infectious diseases caused by bacteria, fungi, and viruses as well as treat non-infectious diseases, such as tumors, cancer, inflammatory responses, and diabetes, and support wound healing. Furthermore, several polymeric materials derived from the marine, such as chitosan and alginate, are exploited as nanocarriers in drug delivery. Moreover, a variety of pure bioactive compounds have been loaded onto polymeric nanocarriers and employed to treat infectious and non-infectious diseases. The current review is focused on a thorough overview of nanoparticle synthesis and its biological applications made from their entire extracts or pure chemicals derived from marine sources.

## 1. Introduction

Infectious diseases have the potential to contribute to an increase in the global death rate [1]. Infectious diseases can be caused by viruses, fungi, and bacteria [2]. These microorganisms cause a variety of diseases, including cholera, candidiasis, and COVID-19 [3,4]. COVID-19 is a recent example that has triggered a pandemic [5]. Multiple drug resistance in viruses, fungi, and bacteria has reached alarming levels that must be addressed promptly. Various health organizations throughout the world have stated that various drug-resistant pathogenic microorganisms must be eradicated quickly [6]. Furthermore, current drugs for treating infectious diseases to patients suffering from non-infectious illnesses, such as cancer, inflammation, obesity, and diabetes, might possibly harm the human body [7,8,9]. To meet this demand, novel molecules that can function as antimicrobials against pathogenic microbes must be investigated [10]. The terrestrial ecosystem has yet to investigate the marine environment [11]. Many applications for marine compounds have been documented [12]. Secondary metabolites produced by marine microorganisms have a wide range of applications [13]. The potential biological activity of marine organisms stems from communication and defensive systems in their natural habitat [13]. Many possible antimicrobial applications from marine sources have been investigated [14].

Furthermore, due to their biodiversity and production of various molecules with varying chemical structures, marine organisms can be exploited as valuable biologics to treat cancer, inflammation, and immune system diseases [15]. As a result of their diverse biological activities, natural compounds derived from marine resources have significantly contributed to disease treatment in place of conventional pharmaceuticals [16]. Nanotechnology is a developing technology with several applications in various sectors [17]. Recent research trends have demonstrated that nanoparticles have a wide range of therapeutic potential [18]. The biosynthesis of nanoparticles is a simple and inexpensive method [19]. Furthermore, the approach of synthesizing nanoparticles from diverse natural products is extensively employed as an eco-friendly method, since it does not produce toxic by-products [20]. Various techniques have been developed to synthesize different types of inorganic nanoparticles, such as gold, zinc, titanium, magnesium, and silver [21]. The biosynthesis of nanoparticles provides antibacterial, drug delivery, sensing, and anticancer treatment. Nanoparticles produced from pure compounds, in particular, outperform traditional drugs in terms of biological activity [22]. This review paper advances our understanding of marine-derived compound nanoparticles as possible therapeutics for a variety of biological roles.

## 2. Green Synthesis of Nanoparticles (NPs) for Its Application in the Field of Medicine

**Figure 1:**
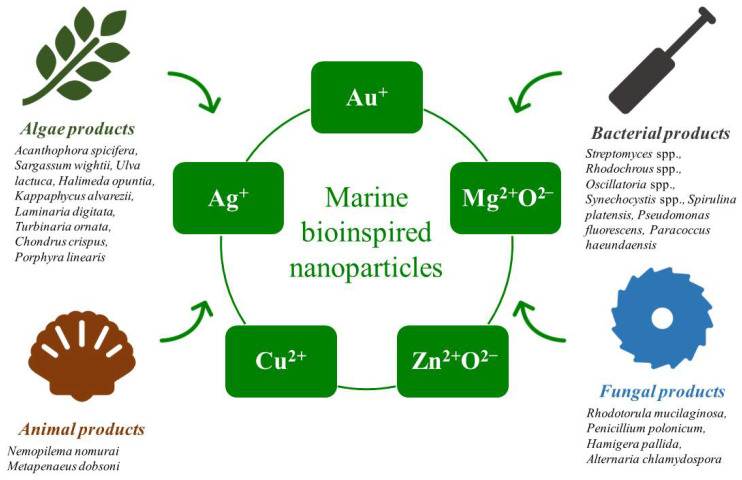
Different types of metal and metal-oxide nanoparticles are synthesized using natural products from various marine organisms.

Nanotechnology is a new discipline of research that works with chemical, biological, and physical sciences to produce nanosized particles with various applications. The size range of nanoparticles has been investigated between 1–100 nm [23,24,25]. Because of their high surface area to volume ratio, nanoparticles have a substantially larger proportion of surface, which leads to enhanced reactivity [26]. Because of their small size, nanoparticles can have a variety of sizes and forms [27]. Nanoparticles have a wide range of applications, including the medicinal, diagnostic, drug discovery, biological sensor, and reagent industries [28]. These biologically active nanoparticles are produced by employing various biological fluids as reducing agents for metal and non-metal ions, such as gold, silver, copper, zinc oxide, platinum, and titanium oxide [29]. The diverse therapeutic applications of nanoparticles, as well as the outbreak of several infectious diseases, motivate this research [30]. The general approaches for nanoparticle production include bioassisted, chemical, and physical methods [31]. Researchers are currently more interested in biological entities than chemical approaches. Fungi, bacteria, plants, and algae from the marine have been found to produce nanoparticles [12]. Green synthetic nanoparticles can be easily decomposed using enzymes included in the nanoparticles, making them more environmentally benign than conventional agents [29]. The reduction of metal ions by reducing agents found in the organism is essential for the synthesis of metal nanoparticles [32]. These reactions are driven by phenolics, terpenoids, alkaloids, amines, carbonyl groups, flavanones, proteins, pigments, and amides found in the organism [33]. Because marine organisms dwell on the unexplored seabed, it is critical to understand the metabolic mechanisms leading to metal ion reduction by diverse types of marine organisms [33]. Figure 1 depicts the different marine organisms, such as algae, bacteria, fungi, and animals, employed in the synthesis of metal nanoparticles.

## 3. Marine Organisms and Compounds for the Green Synthesis of NPs

**Figure 2:**
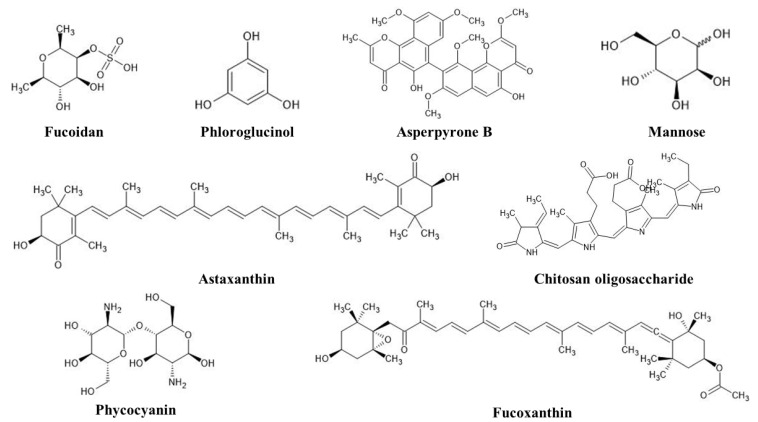
Chemical structures of various pure compounds derived from marine organisms used as reducing agents in nanoparticle synthesis.

Current research and innovation in marine science are contributing to the exponential growth of numerous sectors, including pharmaceuticals, environmental trends, nanomedicine, and food [14]. The ocean covers around 70–71% of the earth’s surface [34]. Previous research studied the oceans, accounting for around 2.2 million distinct species [35]. The ocean contains an unimaginable number of marine-derived compounds with varied applications that are beneficial to humans, such as antimicrobial compounds [36]. The marine ecosystem contains around 25,000 physiologically active chemicals with various applications [37]. Currently, the marine environment paves the way for numerous antibacterial, antifungal, and antiviral compounds. Seaweeds, bacteria, and fungus are possible sources for combating infectious diseases [14,38]. According to prior research, the market for marine-derived compounds has surpassed 10 billion USD [39]. The production of marine-based nanoparticles from a variety of sources, including bacteria, fungus, seaweeds, and marine plants, has received considerable attention [33]. Algae with high cell growth rates, high stress tolerance, and an abundance of physiologically active substances, such as *Ulva lactuca*, *Spirulina platensis*, and *Sargassum muticum*, are regarded as promising biocatalysts for the synthesis of various types of nanomaterials [40,41,42]. Among pure algal compounds, phloroglucinol, eckol, phlorofucofuroeckol A, fucodiphlorethol G, 7-phloroeckol, 6,6′-bieckol, and dieckol act as effective reducing agents in the nanoparticle synthesis process [43]. Diverse marine microorganisms adapt to harsh marine environments as well as a broad variety of temperatures, salinity, and pH, making them suitable biological factories for green nanoparticle synthesis [44]. Bacteria and fungi produce intracellular or extracellular inorganic compounds that react with metal ions to form nanoparticles [45,46]. Furthermore, nanoparticles made from marine-derived animals show good biocompatibility [47]. Seafood waste, in particular, may be used to make a variety of biological products by utilizing its high value-added qualities during the purification process [48]. Figure 2 shows numerous pure compounds obtained from marine organisms that act as reducing agents in the nanoparticle synthesis process.

## 4. Marine Bioinspired NPs Used for Bacterial Infection

Table 1 summarizes a detailed review of marine-based nanoparticles utilized in treating various infectious diseases. Bacterial infection has a negative impact on public health [49]. Nanoparticles are attractive options since they have excellent bactericidal activity when treating pathogenic bacteria [50]. Several studies have been carried out to investigate the mechanisms of marine-inspired nanoparticles as antibacterial agents [51]. In general, marine antimicrobial macromolecules exhibit antibacterial mechanisms, such as (1) inhibition of DNA replication, (2) inhibition of expression of enzymes and other cellular proteins required for ATP production, (3) structural changes and damage to bacterial cell membranes, and (4) ROS production by inhibiting respiratory enzymes [52]. Several marine bacteria, including *Vibrio* spp., *Pseudoalteromonas* spp., and *Ruegeria* spp., generate antimicrobial compounds, a feature seen globally [53]. The marine bacterium *Pseudomonas rhizosphaerae*, in particular, has been shown to produce benzene-type secondary metabolites with potent antibacterial properties [54]. Secondary metabolites produced by marine algae, on the other hand, include polyphenols, terpenes, acetogenin, and aromatic compounds, which have a variety of biological functions, including antibacterial effects [55]. Silver nanoparticles derived from the marine cyanobacterium *Chroococcus minutus* showed antibacterial action against pathogenic strains of *Escherichia coli* and *Streptococcus pyogenes*, which have been discovered to be novel antibacterial for upper respiratory tract infection [56]. The synthesis of silver nanoparticles from cyanobacterium sources had improved control over pathogenic bacteria. Silver nanoparticles derived from the marine endophytic fungus *Penicillium polonicum* showed antibacterial activity against *Acinetobacter baumanii*, with MIC value of 15.62 µg/mL and MBC value of 31.24 µg/mL [44]. These findings were attributed to the activation of apoptosis by altering the osmotic pressure regulation of cells during the interaction of silver nanoparticles and bacteria. Silver nanoparticles using *S. muticum* extracts as a capping agent significantly suppressed the growth of *Bacillus subtilis*, *E. coli*, *Klebsiella pneumoniae*, and *Salmonella* Typhimurium [57]. These silver nanoparticles interacted with the bacterial membrane and penetrated the bacterium. Moreover, silver nanoparticles synthesized using *S. swartzii* showed antibacterial action by producing considerable deterioration in *E. coli* [58]. When combined with silver nanoparticles, *S. wightii* and *Valonopsis pachynema* demonstrated increased antibacterial activity against *Micrococcus luteus* and *S. marcescens* [59]. Silver nanoparticles produced from these seaweeds had a strong antibacterial activity because silver ions caused bacteria to release K^+^ ions. Silver nanoparticles produced from an aqueous extract of *Gelidiella acerosa* inhibited the growth of *P. aeruginosa* and *B. subtilis* [60]. These bacteria were discovered to absorb silver nanoparticles from the cell surface. Silver nanoparticles produced using a culture-free extract of marine *Streptomyces* sp. Al-Dhabi-87 had excellent antibacterial activity against wound-infecting microorganism strains such as *Staphylococcus aureus*, *S. epidermidis*, and *Enterococcus faecalis* [61]. These nanoparticles displayed antibacterial action by releasing intracellular components and altering the cellular structure. Secondary metabolites found in *S. longifolium* extract reduced CuSO_4_ to Cu^2+^, resulting in copper oxide nanoparticles [62]. These CuSO_4_ nanoparticles showed remarkable antibacterial activity against *V. parahemolyticus*, *V. harvey*, *Aeromonas hydrophila*, and *Serratia marcescens*.

**Figure 3:**
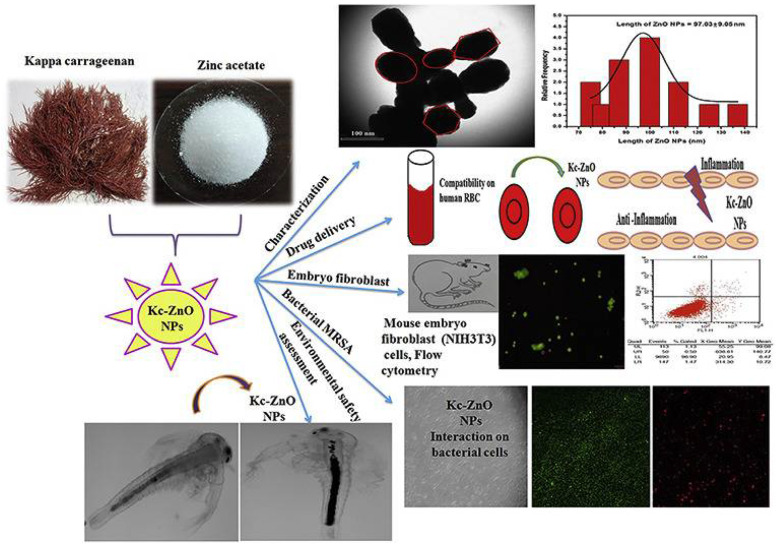
Synthesis of Kappa-Carrageenan wrapped Zinc-oxide nanoparticles (KC-ZnONPs) as an antibacterial agent against Methicillin-resistant *Staphylococcus aureus*. Reprinted with permission from reference [65]. Copyright, 2019 Elsevier B.V.

Carrageenan is a water-soluble, high-molecular-weight, sulfated polysaccharide isolated from many species of red algae. Carrageenan has been widely used in the pharmaceutical, medical, and food industries due to its high viscosity, gelling capacity, and biocompatibility [63,64]. Vijayakumar et al. [65] synthesized Kappa-carrageenan wrapped zinc-oxide nanoparticles (KC-ZnONPs) with antibacterial and antibiofilm activity against Methicillin-resistant *S. aureus* (MRSA) (Figure 3). Based on hemocompatibility studies on human RBCs and eco-safety studies using *Artemia salina*, the synthesized KC-ZnONPs showed high biocompatibility and were non-toxic to the environment.

Fucoidan, a pure chemical derived from *Fucus vesiculosus*, was used to synthesize gold nanoparticles, which demonstrated antibacterial action against *P. aeruginosa* (MIC value of 512 µg/mL) [66]. In addition, the fucoidan-gold nanoparticles reduced the production of virulence factors, such as rhamnolipid, pyocyanin, and pyoverdine. Due to the presence of mannose, the capsular polymeric material isolated from marine *B. altitudinis* proved efficient as a stabilizer for CuO nanoparticle synthesis [67]. The MIC value of CuO nanoparticles containing mannose against *P. aeruginosa* was 1.0 µg/mL. Silver nanoparticles synthesized by the marine fungus *Aspergillus flavus* utilizing amylase showed antibacterial efficacy against Gram-positive and Gram-negative bacteria [68]. In particular, amylase-silver nanoparticles had the strongest antibacterial activity against *A. hydrophila* (MIC value of 1.6 µg/mL). Khan et al. [69] synthesized gold nanoparticles from chitosan oligosaccharide, a natural marine compound, to treat *P. aeruginosa* biofilm infections. Chitosan oligosaccharide-gold nanoparticles exhibited antibiofilm efficacy by lowering bacterial hemolysis and *P. aeruginosa* virulence factors. *P. aeruginosa* hemolysis and protease activity were reduced by a nanocomposite of chitosan and polypyrrole [70]. Moreover, the production of various virulence factors, such as rhamnolipid, pyoverdine, and pyocyanin was reduced by this nanocomposite.

## 5. Marine Bioinspired NPs Used for Fungal Infection

Fungal infection is a constant cause of death [71]. The number of fungal infection cases is increasing, and it has been claimed that over 150 million fungal infections occur yearly, with a 1.5 million death rate from fungal infection [72]. Several secondary metabolites with antifungal action are produced by marine microorganisms, mammals, and algae, similar to antibacterial activity [73]. Antifungal chemicals are produced by a variety of marine species, including bacterial chitinases, lipopeptides, and lactones [74]. Brown algae phlorotannins, on the other hand, have antifungal activity by altering the composition of ergosterol in the yeast cell membrane [75]. The marine depsipeptidepapuamide A has been shown to trigger fungus apoptosis by binding to phosphatidylserine in the cell membrane and entering the plasma membrane [76]. Plakortide F acid, a polyketide endoperoxide produced from marine sponges, also had antifungal activity through affecting Ca^2+^ homeostasis [77]. As a result, many researchers continue to look for antifungal activity in a variety of marine organisms for application to nanoparticles. Green synthesis of silver nanoparticles from *U. rigida* had antifungal action against the fungus *Trichophyton mantigrophytes* and *T. cutaneum*, which are linked toskin infections [78].

**Table 1:** List of marine-bioinspired metallic nanoparticles treating infectious diseases.

Name of Marine-Derived Compound/Product	Sources/Organism	Name of NPs	Size Range of MNPs	Shape/Morphology	Antimicrobial Types	Microbial Pathogens	References
Extracts	*Ulva rigida* *Cystoseira myrica* *Gracilaria foliifera*	AgNPs	12 nm	Spherical	Antibacterial	*Bacillus cereus* *Escherichia coli* *Candida albicans* *Staphylococcus aureus* *Cryptococcus neoformans*	[78]
Extracts	*U. lactuca*	AgNPs	20–50 nm	-	Antibacterial	*E. coli* *Enterobacter* spp. *Klebsiella pneumonia* Methicillin-resistant *S. aureus* (MRSA) *Pseudomonas aeruginosa* *S. aureus*	[79]
Extracts	*Halimeda opuntia* *Kappaphycus alvarezii*	SeNPs	30, 80 nm	Spherical	Antibacterial	*Vibrio harveyi* *V. parahaemolyticus*	[80]
Extracts	*Spirulina platensis*	SNPs	200–450 nm	Spherical	Antibacterial	*V. parahaemolyticus*	[81]
Extracts	*Chroococcus minutus*	AgNPs	-	-	Antibacterial	*E. coli* *Streptococcus pyogenes*	[56]
Extracts	*U. lactuca*	SeNP	85 nm	Spherical	Antibacterial	*Lactobacillus* *C. albicans* *S. mutans* *S. aureus*	[82]
Extracts	*Sargassum muticum*	AgNPs	20–54 nm	Spherical	Antibacterial	*E. coli* *B. subtilis* *Salmonella* Typhimurium *K. pneumoniae*	[57]
Extracts	*S. swartzii*	AgNPs	20–40 nm	Spherical	Antibacterial	*E. coli*	[58]
Extracts	*Gelidium corneum*	AgNPs	20–50 nm	Spherical	Antibacterial	*E. coli*	[83]
Extracts	*Laminaria ochroleuca*	AgNPs	10–20 nm	Spherical	Antibacterial	*E. coli* *B. cereus* *P. aeruginosa* *S. aureus* *K. pneumoniae* *Micrococcus luteus*	[84]
Extracts	*Streptomyces* sp. Al-Dhabi-87	AgNPs	10–17 nm	Spherical	Antibacterial	*Enterococcus faecalis* *E. coli* *S. aureus* *S. epidermidis* *P. aeruginosa* *K. pneumoniae*	[61]
Extracts	*S. wightii* *Valonopsis pachynema*	AgNPs	30–40, 55–70 nm	-	Antibacterial	*M. luteus* *Serratia marcescens*	[59]
Extracts	*Streptomyces* sp. *Rhodococcus rhodochrous*	AgNPs	5.52, 35 nm	Spherical	Antibacterial	*B. subtilis* *E. coli* *P. aeruginosa* *S. aureus*	[85]
Extracts	*Gelidiella acerosa*	AgNPs	-	-	Antibacterial	*B. subtilis* *P. aeruginosa* *S. aureus*	[60]
Extracts	*Acanthophora spicifera*	AuNPs	<20 nm	Spherical	Antibacterial	*V. harveyi* *S. aureus*	[86]
Extracts	*G. amansii*	AgNPs	27–54 nm	Spherical	Antibacterial	*Aeromonas hydrophila* *V. parahaemolyticus* *P. aeruginosa* *E. coli* *S. aureus* *B. pumilus*	[87]
Extracts	*S. wighitii*	MgONPs	68.06 nm	Flower	Antibacterial	*S. pneumonia* MRSA *E. coli* *P. aeruginosa* *A. baumannii*	[88]
Extracts	*Oscillatoria princeps*	AgNPs	3.30–17.97 nm	Spherical	Antibacterial	*S. aureus* *S. pyogenes* *E. coli*	[89]
Extracts	*Nocardiopsis dassonvillei*-DS013	AgNPs	30–80 nm	Circular	Antibacterial	*Enterococcus* sp. *Klebsiella* sp. *B. subtilis* *Streptococcus* sp. *Proteus* sp. *Pseudomonas* sp. *Shigella* sp. *E. coli*	[90]
Extracts	*Streptomyces* sp. Al-Dhabi-87	AgNPs	11–21 nm	Cubic	Antibacterial	*B. subtilis* *S. aureus* *S. epidermidis* *P. aeruginosa* *E. coli* *E. faecalis* *K. pneumoniae*	[91]
Extracts	*Penicillium polonicum*	AgNPs	10 nm	Spherical	Antibacterial	*A. baumanii*	[44]
Chitosan	Marine Seafood	AgNPs	5–20 nm	Spherical	Antibacterial	*E. coli* *P. aeruginosa*	[92]
Chitosan	*Aspergillus* sp. *Alternaria* sp.	Chitosan-AgNPs Chitosan-AuNPs	4.5 ± 20.0–50.2 ± 74.0 nm 3.47 ± 2.00–35.50 ± 2.00 nm	Spherical	Antibacterial	*S. aureus*	[93]
Extracts	*Chlorococcum humicola* *Chlorella vulgaris*	AgNPs	10.69, 12.83 nm	Spherical	Antibacterial	*E. coli* *S.* Typhimurium *K. pneumoniae*	[94]
Extracts	*Cymodocea serrulata*	AgNPs	40.49–66.44 nm	-	Antibacterial	*V. parahaemolyticus*	[95]
Extracts	*S. longifolium*	CuONPs	40–60 nm	-	Antibacterial	*V. parahemolyticus* *A. hydrophila* *Serratia marcescens* *V. harveyi*	[62]
Extracts	*C. crinita*	ZnONPs	23–200 nm	Rectangular	Antibacterial	*E. coli* *B. cereus* *S.* Typhimurium *S. aureus* *C. albicans* *A. niger*	[96]
Extracts	*Synechocystis* sp.	AgNPs	10–35 nm	Spherical	Antibacterial	MRSA	[97]
Extracts	*O. limnetica*	AgNPs	3.30–17.97 nm	Quasi-spherical	Antibacterial	*E. coli* *B. cereus*	[98]
Extracts	Red algae	Co_3_O_4_NPs	29.8 ± 8.6 nm	Spherical	Antibacterial	*P. aeruginosa* *E. coli*	[99]
Extracts	*U. lactuca*	AgNPs	20–50 nm	-	Antiviral	*Aedes aegypti* *Culex pipiens*	[79]
Extracts	*Oscillatoria* sp. *S. platensis*	Ag_2_O/AgONPs AuNPs	14.42–48.97 nm 15.60–77.13 nm	Spherical Octahedral, pentagonal and triangular	Antiviral	HSV-1	[100]
Extracts	*Streptomyces* sp. *R. rhodochrous*	AgNPs	5.52–35.00 nm	Spherical	Antiviral	Poliovirus	[85]
Extracts	*Pectinodesmus* sp. strain HM3 *Dictyosphaerium* sp. strain HM1 *Dictyosphaerium* sp. strain HM2	AgNPs	50–65, 15–30, and 40–50 nm	Spherical	Antiviral	Newcastle disease virus	[101]
Extracts	*U. rigida*	AgNPs	12 nm	Spherical	Antifungal	*Trichophyton mentagrophytes* *T. cutaneum*	[78]
Extracts	*S. griseus*	AgNPs	14.54 nm	Spherical	Antifungal	*C. albicans*	[102]
Extracts	*G. corneum*	AgNPs	20–50 nm	Spherical	Antifungal	*C. albicans*	[83]
Extracts	*P. fluorescens*	AgNPs	-	-	Antifungal	*Fusarium udum* *A. niger*	[103]
Extracts	*C. serrulate* *Padina australis*	AgNPs	-	-	Antifungal	*Pyriporia oryzea* *Helminthosporium oryzae* *Alternaria* sp. *Rhizoctonia solani* *Xanthomoanas oryzae*	[104]
Extracts	*C. umhumicola* *C. vulgaris*	AgNPs	10.69,12.83 nm	Spherical	Antifungal	*F. solani* *F. moniliforme* *Penicillium* sp.	[94]

**Figure 4:**
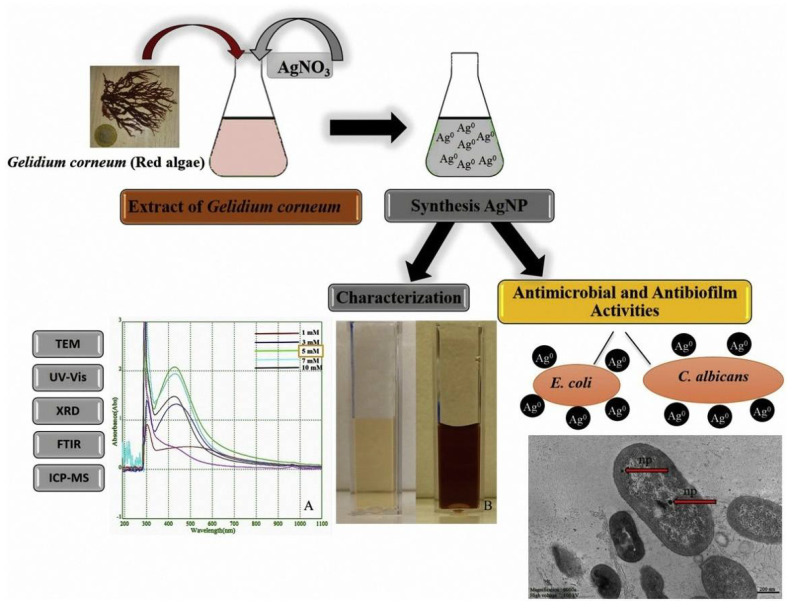
Synthesis and characterization of AgNPs using extract of marine red algae *Gelidium corneum* with antimicrobial and antibiofilm inhibition characteristics towards *Escherichia coli* and *Candida albicans*. (**A**) UV-vis absorption spectra of AgNPs synthesized using extract in the presence of different silver salt concentrations and (**B**) Change in color of the reaction mixture indicates the formation of AgNPs. Reprinted with permission from reference [83]. Copyright, 2019 Elsevier Ltd.

These silver nanoparticles produced an insoluble chemical that inactivated the fungal cell wall’s sulfhydryl group and disrupted the membrane, resulting in an antifungal effect. Silver nanoparticles synthesized from aqueous extracts of *Cymodocea serrulata* and *Padina australis* had antifungal action against plant fungi, including *Pyriporia oryzea*, *Alternaria* sp., *Helminthisporium oryzea*, *Rhizoctonia solani*, and *Xanthomanas oryzae* [104]. These antifungals were discovered as a consequence of cell wall disruption, DNA damage, and an increase in ROS. Silver nanoparticles (AgNPs) synthesized from *Gelidium corneum* extract, which served as a reducing agent had excellent antifungal and antibiofilm properties against *Candida albicans* [83]. Biosynthetic silver nanoparticles, in particular, demonstrated antifungal effectiveness by generating cell membrane and cell wall destruction, as well as cytoplasmic damage (Figure 4).

## 6. Marine Bioinspired NPs for Treating Viral Infection

A viral particle is smaller than a live cell. Several viral infections have been documented to be caused by a pathogenic virus. Viruses cause a variety of diseases, leading to increased death rate. Viral infections include smallpox, polio, HIV, and hepatitis C [105]. Antiviral compounds produced by marine organisms include polyphenols, alkaloids, lipids, carbohydrates, steroids, terpenoids, exopolysaccharides, polyketides, zoanthoxanthins, and peptides [106]. Virus adsorption, penetration, capsid decoration, biosynthesis, virus assembly, and virus release are all inhibited or inactivated by marine polysaccharides [107]. One of the metabolites produced by marine organisms, phlorotannins, has been shown to interfere with viral attachment, penetration, and replication [15]. Silver nanoparticles derived from the seaweed *U. lactuca* showed cytotoxic efficacy against the vector-borne pathogens *Aedes aegypti* and *Culex pipiens* [79]. Because of their small particle size, silver nanoparticles synthesized from *U. lactuca* demonstrated more effective action than conventional insecticides. Additionally, these silver nanoparticles bonded to the insect cuticle and entered within the cell, disrupting additional cell functions. Silver nanoparticles mediated by *Oscillatoria* sp. and gold nanoparticles mediated by *S. platensis* displayed antiviral efficacy against herpesvirus [100]. These nanoparticles induced glycoprotein aggregation and surface changes, both of which might inhibit viral binding and penetration. Silver nanoparticles derived from extracellular extracts of the marine actinomycetes *Rhodococcus rhodochrous* and *Streptomyces* sp. inhibited poliovirus in RD cells [85]. The interaction of viral proteins with silver nanoparticles caused poliovirus inhibition. Silver nanoparticles derived from *Dictyosphaerium* sp., a freshwater microalgae, had substantial antiviral activity against the Newcastle disease virus [101]. These silver nanoparticles were bound to the viral glycoprotein envelope, limiting virus penetration.

## 7. Marine Bioinspired NPs for Treating Non-Infectious Diseases

**Figure 5:**
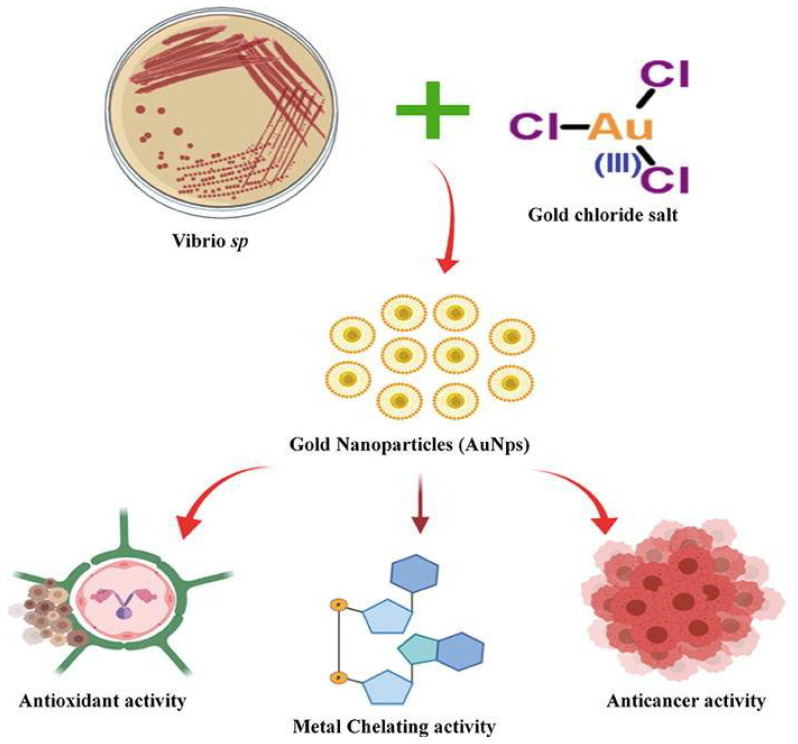
Anticancer and antioxidant properties of gold nanoparticles synthesized using marine microbe *Vibrio alginolyticus*. Reprinted with permission from reference [120]. Copyright 2020, Elsevier B.V.

Table 2 shows studies that have used marine bioinspired nanoparticles to treat a wide range of non-infectious diseases. Inflammation is the body’s natural response to tissue injury, infection, and genetic alterations [108]. The immune system is activated within the body under inflammatory circumstances, resulting in the release of various inflammatory mediators, such as bradykinins and prostaglandins [109]. Thus, reducing prostaglandin levels can aid in the prevention of chronic disease by controlling inflammation [110]. To reduce inflammation and inflammatory mediators, steroids and nonsteroidal antiinflammatory medications constitute one of the treatment paths for inflammatory diseases [111]. These synthetic antiinflammatory drugs, on the other hand, can have substantial negative effects [112]. As a result, it is required to use marine-based organisms with high biological activity to generate nanoparticles with similar therapeutic benefits and no negative effects. Silver nanoparticles produced from macroalgae such as *Galaxaura elongate*, *Turbinaria ornate*, and *Enteromorpha flexuosa* have shown considerable antiinflammatory action via membrane stabilization [113]. Silver nanoparticles, in particular, reduced prostaglandin production by inhibiting protein denaturation, cyclooxygenase, and 5-lipoxygenase. At 500 µg/mL, ZnO nanoparticles wrapped in Kappa-carrageenan demonstrated 82% antiinflammatory efficacy (Figure 3) [65]. Because of their high surface area to volume ratio, these nanoparticles were more effective than bulk materials in inhibiting cytokines and inflammatory coenzymes. Cancer is caused by the uncontrollable growth of cells and tissues [114]. Cancer treatment options include surgery, radiation, and potentially toxic medication therapy [115]. As a result, several investigations are being done to discover anticancer drugs that kill cancer cells without hurting humans [116]. Nanoparticles loaded with various physiologically active chemicals are one of the most effective drug delivery techniques for cancer therapy [117]. Marine-derived natural products, in particular, are potential molecules for the development of anticancer drugs because they may influence multiple pathways, such as immunity, cancer cell death, and tumor growth [118]. Silver nanoparticles derived from *Caulerpa taxifolia* showed antitumor efficacy against A549 lung cancer cells [119]. Necrosis and condensation of A549 cells were shown to be mediated by silver nanoparticles derived from marine algae, suggesting that nanomaterials are relevant for cancer cell research. Furthermore, gold nanoparticles inhibited phosphorylation of AKT and ERK, which are essential for cell growth in HeLa cancer cells [47]. Interestingly, these gold nanoparticles derived from jellyfish extract exhibited a significant lethal effect on HeLa cancer cells. Cu_2_O nanoparticles derived from *Rhodotorula mucilaginosa* showed anticancer activity against SKOV-3, MCF-7, HepG2, A549, SW620, and HT-29 [117]. In particular, reactive oxygen species production and oxidative stress enhanced the anticancer mechanism of Cu_2_O nanoparticles. Similarly, Shunmugam et al. [120] synthesized gold nanoparticles from the marine bacterium *V. alginolyticus*, which had antioxidant and anticancer activity (Figure 5). The anticancer activity was attributed to the treated cells’ nuclear condensation.

Silver nanoparticles derived from shrimp shell chitin acted as a reducing agent and had anticancer action against human hepatocarcinoma [129]. In HepG2 cells, chitin-silver nanoparticles increased the expression of apoptosis-related proteins Bax, PARP, cytochrome-c, caspase-3, and caspase-9, while decreasing the expression of antiapoptosis proteins Bcl-2 and Bcl-xl. Phloroglucinol-encapsulated starch biopolymer exhibited dose-dependent anticancer activities against the HepG2 liver cancer cell line [130]. These findings were ascribed to the biopolymer’s hydrophobicity, which increased adhesion and adsorption capability to the cancer cell surface.

Cells produce potentially harmful ROS as a result of oxygen metabolism, which involves enzymatic and non-enzymatic reactions [131]. High levels of ROS caused by oxidative stress induce a variety of diseases in the body, including diabetes, hypertension, and Alzheimer’s [132]. Antioxidants, on the other hand, have a role in delaying, regulating, and avoiding the oxidative process that leads to the beginning and progression of the disease [133]. Through SOD enzymes, which catalyze the recombination of oxygen radicals, these antioxidants counteract the consequences of oxidative stress [134]. Currently, research is being performed to investigate natural substances capable of controlling oxidative stress, which leads to the investigation of nanoparticles with antioxidant activities. Many species with antioxidant activity in marine organisms, in particular, have been found and have piqued the interest of researchers due to their potential biological activity [135]. The antioxidant activity of gold nanoparticles produced by the marine fungus *A. chlamydospora* (inhibition of DPPH radicals) was dose-dependent [125]. Furthermore, in a concentration-dependent way, gold nanoparticles mediated by the marine bacteria *Paracoccus haeundaensis* cell-free supernatant demonstrated strong reducing power via DPPH scavenging activity [128]. Selenium nanoparticles produced from *Spirulina* phycocyanin protected INS-1E rat insulinoma cells against palmitic acid-induced cell death [136]. Phycocyanin and selenium shielded cells from oxidative damage and signaling pathways downstream. These findings indicate that marine-derived nanoparticles can be employed as effective natural antioxidants.

**Figure 6:**
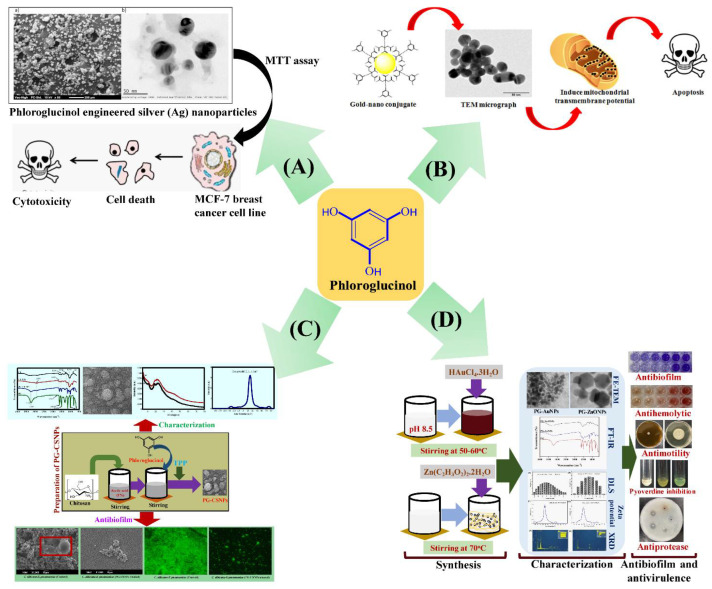
Application of phloroglucinol in the form of nanoparticles for treating infectious and non-infectious diseases. (**A**) The cytotoxicity action of phloroglucinol-engineered AgNPs towards MCF-7 breast cancer cell lines. (**a**) SEM image of AgNPs and (**b**) TEM image of AgNPs. Reproduced with permission from reference [137]. (**B**) Synthesis of the phloroglucinol-conjugated gold nanoparticles, which exhibit therapeutic potential towards cancer cells. The action mechanism involved apoptosis of cancer cells by promoting mitochondrial transmembrane permeation, as evident by fluorescence staining and gene expression studies. Reprinted with permission from reference [138], (**C**) Encapsulation of phloroglucinol into the chitosan nanoparticles. The PG-CSNPs exhibit antibiofilm properties towards single- and mixed-species biofilms of *C. albicans*-*S. aureus*/*S. mutans*/*K. penumoniae*. Reprinted with permission from reference [139], and (**D**) Synthesis of metal (AuNPs) and metal oxide (ZnONPs) nanoparticles using phloroglucinol. The synthesized PG-AuNPs and PG-ZnONPs showed antibiofilm and antivirulence properties towards *P. aeruginosa*. Reproduced with permission from reference [140]. Copyright 2021 by the authors and licensee MDPI, Basel, Switzerland.

Figure 6 depicts the biological activity of nanoparticles synthesized using phloroglucinol. Silver nanoparticles synthesized using phloroglucinol showed anticancer efficacy against the MCF-7 breast cancer cell line [137]. Silver ions from phloroglucinol silver nanoparticles entered cancer cells and interacted with intracellular macromolecules, such as organelles, proteins, and DNA, to trigger apoptosis. Another study found that phloroglucinol-gold nanoparticles triggered death in HeLa cancer cells via enhancing mitochondrial membrane permeability [138]. Phloroglucinol-encapsulated chitosan nanoparticles showed antibiofilm action against single-species biofilms, such as *K. pneumoniae*, *S. aureus*, *S. mutans*, and *C. albicans,* and mixed-species biofilms, such as *C. albicans*-*S. aureus/K. pneumoniae*/*S. mutans* [139]. Gold and zinc oxide nanoparticles produced with phloroglucinol showed significant antibacterial action against *P. aeruginosa* [140]. Moreover, these nanoparticles inhibited *P. aeruginosa* twitching, swimming, and swarming motility, all of which have virulence features. Similarly, several marine-derived pure compounds are employed in synthesizing nanoparticles and encapsulating drugs for application in the field of medicine (Table 3).

## 8. Conclusions and Future Perspectives

In conclusion, because of their potential biological activity, marine-derived products have been widely used in the pharmaceutical industry. With an increased understanding of their biological functions, various marine-derived compounds have been used in the synthesis of nanoparticles. These products comprised polymers, organic compounds, and extract, which act as a powerful reducing agent in synthesizing metal and metal-oxide nanoparticles. Furthermore, certain polymeric material is used to effectively deliver the drug in the treatment of infectious and non-infectious diseases. The review detailed the list of the marine organism from which the extract was extracted and which was used to synthesize several forms of nanoparticles. Furthermore, these nanoparticles have been shown to have antimicrobial properties against bacterial, fungal, and viral pathogens. Antimicrobial mechanisms include the breakdown of cell membranes, as well as damage to cell walls and DNA. These marine-inspired nanoparticles have also shown promise in the treatment of non-infectious diseases, such as diabetes, cancer, wounds, inflammatory reactions, and leishmanial infections. Though significant progress has been made in the production of nanoparticles utilizing extracts from marine sources, relatively little information is known on the synthesis of nanoparticles using pure active compounds. This is because of the fact that there are several variations in extract preparation due to a number of environmental factors. As a result, future research should prioritize the use of pure active compounds for nanoparticle synthesis. Most antimicrobial research involving these nanoparticles has been conducted at the phenotypic level; however, investigations at the gene level are highly needed to explain the molecular mechanism.

## Figures and Tables

**Table 2:** List of marine-bioinspired metallic nanoparticles for treating non-infectious diseases.

Name of Marine-Derived Compound/Product	Organisms/Sources	Name of NPs	Size Range of MNPs	Shape/Morphology	Types of Non-Infectious Disease Treatment	Effects/Activities	References
Extracts	*Ulva rigida* *Cystoseira myrica* *Gracilaria foliifera*	AgNPs	12 nm	Spherical	Anticancer	Human breast adenocarcinoma cell line	[78]
Extracts	*Streptomyces* sp. *Rhodococcus rhodochrous*	AgNPs	5.52, 35 nm	Spherical	Anticancer Anti-leishmanial	HepG2 cell line *Leishmania tropica*	[85]
Extracts	*Acanthophora spicifera*	AuNPs	<20 nm	Spherical	Anticancer	Human colon adenocarcinoma (HT-29) cells	[86]
Extracts	*Sargassum wighitii*	MgONPs	68.06 nm	Flower	Anticancer	A549	[88]
Extracts	*Rhodotorula mucilaginosa*	Cu_2_ONPs	51.6–111.4 nm	Spherical	Anticancer	SW620 SKOV-3 MCF-7 HT-29 HepG2 A549	[117]
Extracts	*Pterocladia capillacea*	CuONPs	62 nm	Spherical	Anticancer	Breast cancer, ovarian cancer, and hepatocellular carcinoma cell lines	[121]
Extracts	*Laminaria digitata*	ZnONPs	100–350 nm	Spindle	Anticancer	Fibroblasts cells and human colon cancer cells	[122]
Extracts	*Hamigera pallidass*	AgNPs	5.85 ± 0.84, 3.69–16.11 nm	Spherical	Anticancer Antioxidant	Human breast cancer DPPH (2, 2-diphenyl1-picrylhydrazyl)	[123]
Extracts	*Galaxaura elongate* *Turbinaria ornata* *Enteromorpha flexuosa*	AgNPs	30–90, 20–60, 30–90 nm	Spherical	Anticancer Antioxidant Antidiabetic Antiinflammatory	HepG2 DPPH, ABTS scavenger α-Amylase inhibition Proteinase inhibition and albumin denaturation inhibition	[113]
Extracts	*U. lactuca*	AgNPs	8–14 nm	Spherical	Anticancer	Human colon cancer	[124]
Extracts	*Alternaria chlamydospora*	AuNPs	-	Spherical	Anticancer Antioxidant	A549 cell lines DPPH	[125]
Extracts	*Chondrus crispus* *Gelidium corneum* *Porphyra linearis*	AuNPs	16.9 ± 2.5, 15.0 ± 3.0, 44.2 ± 6.1 nm	Spherical	Antitumoral Antioxidant	Monocytic cell line Human promyelocytic cells	[126]
Extracts	*C. crinita*	ZnONPs	23–200 nm	Rectangular	Antioxidant	DPPH	[96]
Extracts	*Synechocystis* sp.	AgNPs	10–35 nm	Spherical	Wound-healing	Diabetic wounded animals	[97]
Carrageenan &Carrageenan oligosaccharide	Marine red algae	AuNPs	141 ± 6 nm	Spherical	Anticancer	HCT-116 and HepG2 cells	[127]
Extracts	*Paracoccus haeundaensis*	AuNPs	20.93 ± 3.46 nm	Spherical	Anticancer Antioxidant	A549 AGS cancer cells DPPH	[128]
Extracts	*Caulerpa taxifolia*	AgNPs	-	-	Anticancer	A549 lung cancer cells	[119]
Extracts	*Nemopilema nomurai*	AuNPs	35.2 ± 8.7 nm	Spherical	Anticancer	HeLa cancer cells	[47]
Extracts	*Oscillatoria limnetica*	AgNPs	3.30–17.97 nm	Quasi-spherical	Anticancer	Human colon cancer cell line Human breast cell line	[98]
Extracts	Red algae	Co_3_O_4_NPs	29.8 ± 8.6 nm	Spherical	Anticancer	HepG2 cancer cells	[99]
Extracts	*Vibrio alginolyticus*	AuNPs	50–100 nm	Monodispersed, irregular shape	Anticancer	HCA-7 cells	[120]

**Table 3:** Application of marine-derived compounds in the synthesis of nanoparticles and encapsulation of drugs for application in the field of medicine.

Classification of Sources	Natural Pure Compounds	Types of Nanomaterial	Size	Morphology	Biological Activity	Action Mechanism	References
Algae	Fucoidan	AuNPs	~53 nm	Spherical	Antibacterial activity against *Pseudomonas aeruginosa*	Reduced the generation of numerous important virulence factors Impaired bacterial motility, including twitching, swimming, and swarming	[66]
Algae	Phloroglucinol	AuNPs and ZnONPs	41.6 ± 3.9, 52.7 ± 3.8 nm	Spherical and hexagonal	Antibacterial activity against *P. aeruginosa*	*P. aeruginosa* virulence factors, such as rhamnolipid, pyocyanin, pyoverdine, protease, and hemolytic capabilities, were reduced. Impaired bacterial motility, including twitching, swimming, and swarming	[140]
Algae	Phycocyanin	SeNPs	165, 235, 371, 815 nm	Spherical	Antioxidant	Protected INS-1E cells against palmitic acid-induced cell death by reducing oxidative stress and signaling pathways downstream	[136]
Algae	Fucoxanthin	AgNPs	20–25 nm	Spherical	Antibacterial activity against *Escherichia coli*, *Bacillus stearothermophilus*, and *Streptococcus mutans*	-	[141]
Algae	Phloroglucinol	Starch biopolymer	1–100 nm	Spherical	Anticancer	Adhesion and adsorption on the surfaces of cancer cells are enhanced	[130]
Algae	Phloroglucinol	CSNPs	414.0 ± 48.5 nm	Spherical	Antibiofilm activity against *Klebsiella pneumoniae*, *Staphylococcus aureus*, *Candida albicans*, *S. mutans*, and mixed-species such as *C. albicans*-*S. aureus*/*K. pneumoniae*/*S. mutans*	The positive charge of CSNPs allows for easy biofilm penetration and binding	[139]
Algae	Usnic acid	Nanofibrous poly(ε-caprolactone)/decellularized extracellular matrix scaffolds	3.89 ± 2.52, 4.95 ± 2.19, 5.00 ± 2.05 μm	Fusion of the fiber junctions	Antibacterial activity against *Cutibactrium acnes*, *S. mutans*, *S. aureus*, *S. epidermidis*, and *C. albicans* Antibiofilm activity against *K. pneumoniae* and *P. aeruginosa* Wound healing capability	Increased swelling, surface erosion, and degradation due to high release qualities Improved cellular activities, such as cell adhesion, proliferation, differentiation, and migration	[142]
Algae	Carrageenan	ZnONPs	97.03 ± 9.05 nm	Hexagonal wurtzite phase	Antibacterial activity against MRSA Antiinflammatory activity	Penetrated quickly through the bacterial cell membrane and had a greater bactericidal impact Inflammation enhancers such as cytokines and inflammation-assist enzymes are blocked	[65]
Bacteria	Mannose	CuONPs	108 nm	Spherical	Antibacterial activity against *P. aeruginosa*	Entered the cell membrane, causing lysis and cell rupture	[67]
Fungi	Asperpyrone B Asperpyrone C	AgNPs	8–30 nm	Spherical	Acetylcholine esterase inhibitory activity	Enzyme structural alterations	[143]
Fungi	α-amylase	AgNPs	22.88–26.35 nm	Spherical	Antibacterial activity against *Aeromonas hydrophila*, *P. aeruginosa*, *Vibrio anguillarum*, *S. faecium*, *S. agalactiae*, and *Listeria* spp.	Damage to cell membranes, oxidative stress, and protein and DNA damage	[68]
Animal	Chitin	AgNPs	17–49 nm	Spherical	Anticancer activity in human hepatocellular carcinoma HepG2 cells	Increased levels of apoptosis-related proteins, such as PARP, cytochrome-c, Bax, caspase-3, and caspase-9 Reduced expression of the antiapoptotic proteins Bcl-xL and Bcl-2 in HepG2 cells	[129]
Animal	Astaxanthin	AuNPs	58.2 ± 4.6 nm	Polygonal and spherical	Antioxidant	Reduced ROS and increased antioxidant enzyme activity in rice plants treated to Cd to alleviate oxidative stress	[144]
Animal	Chitosan oligosaccharide	AuNPs	56.01 ± 3.48 nm	Spherical	Antibacterial activity against *P. aeruginosa*	Inhibited bacterial hemolysis Reduced *P. aeruginosa* virulence factor synthesis Reduced bacterial swimming and twitching motilities	[69]
Animal	Thiol chitosan	AuNSs	185 ± 19 nm	Spherical	Antibacterial activity against *E. coli, P. aeruginosa,* and *S. aureus*	-	[145]
Animal	Chitosan	Polypyrrole nanocomposites	55.77 ± 3.48 nm	Spherical	Antibiofilm activity against *P. aeruginosa*	*P. aeruginosa* hemolytic and protease activities were inhibited Reduced the production of many virulence factors, including pyocyanin, pyoverdine, and rhamnolipid	[70]

## Data Availability

Not applicable.
